# Tanshinone I specifically suppresses NLRP3 inflammasome activation by disrupting the association of NLRP3 and ASC

**DOI:** 10.1186/s10020-023-00671-0

**Published:** 2023-07-03

**Authors:** Jia Zhao, Hongbin Liu, Zhixian Hong, Wei Luo, Wenqing Mu, Xiaorong Hou, Guang Xu, Zhie Fang, Lutong Ren, Tingting Liu, Jincai Wen, Wei Shi, Ziying Wei, Yongping Yang, Wenjun Zou, Jun Zhao, Xiaohe Xiao, Zhaofang Bai, Xiaoyan Zhan

**Affiliations:** 1 Department of Hepatology, the Fifth Medical Center of Chinese PLA General Hospital, Beijing, 100039 China; 2 School of Pharmacy, Chengdu University of Traditional Chinese Medicine, Chengdu, 611137 China; 3 School of Pharmacy, North SiChuan Medical College, Nanchong, 637000 China; 4 Department of Pharmacy, Hebei Key Laboratory of Neuropharmacology, Hebei North University, Zhangjiakou, 075000 China

**Keywords:** Tanshinone I, NLRP3 inflammasome, ASC, LPS-induced septic shock, NASH

## Abstract

**Background:**

Abnormal activation of NLRP3 inflammasome is related to a series of inflammatory diseases, including type 2 diabetes, gouty arthritis, non-alcoholic steatohepatitis (NASH), and neurodegenerative disorders. Therefore, targeting NLRP3 inflammasome is regarded as a potential therapeutic strategy for many inflammatory diseases. A growing number of studies have identified tanshinone I (Tan I) as a potential anti-inflammatory agent because of its good anti-inflammatory activity. However, its specific anti-inflammatory mechanism and direct target are unclear and need further study.

**Methods:**

IL-1β and caspase-1 were detected by immunoblotting and ELISA, and mtROS levels were measured by flow cytometry. Immunoprecipitation was used to explore the interaction between NLRP3, NEK7 and ASC. In a mouse model of LPS-induced septic shock, IL-1β levels in peritoneal lavage fluid and serum were measured by ELISA. Liver inflammation and fibrosis in the NASH model were analyzed by HE staining and immunohistochemistry.

**Results:**

Tan I inhibited the activation of NLRP3 inflammasome in macrophages, but had no effect on the activation of AIM2 or NLRC4 inflammasome. Mechanistically, Tan I inhibited NLRP3 inflammasome assembly and activation by targeting NLRP3-ASC interaction. Furthermore, Tan I exhibited protective effects in mouse models of NLRP3 inflammasome-mediated diseases, including septic shock and NASH.

**Conclusions:**

Tan I specifically suppresses NLRP3 inflammasome activation by disrupting the association of NLRP3 and ASC, and exhibits protective effects in mouse models of LPS-induced septic shock and NASH. These findings suggest that Tan I is a specific NLRP3 inhibitor and may be a promising candidate for treating NLRP3 inflammasome-related diseases.

**Graphical Abstract:**

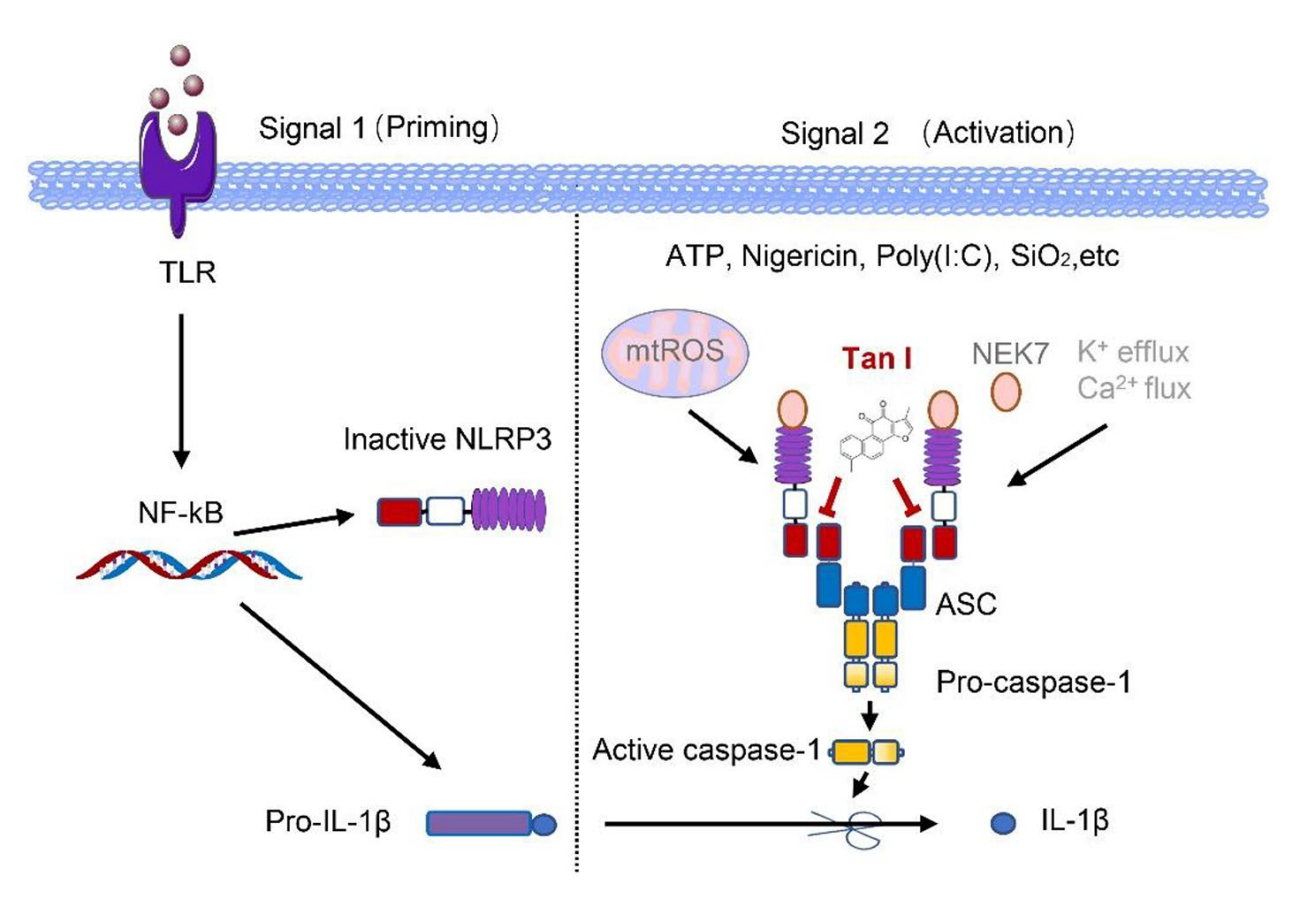

**Supplementary Information:**

The online version contains supplementary material available at 10.1186/s10020-023-00671-0.

## Introduction

Inflammasome, a cytosolic multi-protein complex, composed of effector molecule caspase-1, the adaptor protein apoptosis-associated speck-like protein (ASC), as well as recognition molecules (Rathinam and Fitzgerald 2016). The recognition molecules include a variety of NOD-like family molecules (NLRs), including NLRP1, NLRP3, NLRC4 (Elliott and Sutterwala 2015), and PYHIN 8 family member absent in melanoma 2 (AIM2) (Lugrin and Martinon 2018). NLPR3 inflammasome can be activated by both pathogen-associated molecular patterns (PAMPs) and damage-associated molecular patterns (DAMPs) (Kelley et al. 2019). Once activated, NLRP3 forms inflammasome together with ASC and pro-caspase-1, acting as the platform for caspase-1 activation, resulting in the secretion of IL-18 and IL-1β, which mediate the progression of multiple inflammatory diseases (Duez and Pourcet 2021; Youm et al. 2015).

Since NLRP3 can sense pathogens and many stimuli derived from the host, abnormal NLRP3 inflammasome activation is demonstrated to be an essential driver of a series of complex human diseases, including acute kidney injury, atherosclerosis, non-alcoholic steatohepatitis (NASH), and type 2 diabetes (Li et al. 2022a, b; Wang et al. 2021; Yang et al. 2022; Zhao et al. 2021), suggesting that NLPR3 inflammasome may act as a potential therapeutic target for these diseases (Chauhan et al. 2020; Wang and Hauenstein 2020). Therefore, inhibiting NLRP3 inflammasome may be a promising approach for treating NLRP3-mediated inflammatory diseases (Chiazza et al. 2016; Pappritz et al. 2022; Swanson et al. 2019).

Increasing evidence suggests that small molecule compounds from traditional Chinese medicine targeting NLRP3 inflammasome, such as oridonin from *Rabdosia rubescens* (He et al. 2018), cardamonin from *Alpinia katsumadai* (Wang et al. 2019b), echinatin from *Glycyrrhiza plants* (licorice) (Xu et al. 2021), exhibit therapeutic effects on NLRP3-mediated disease in animal models. Tanshinone I (Tan I), the active ingredient in the traditional Chinese medicine *Salvia miltiorrhiza* (Zhou et al. 2020). Recent reports have demonstrated its multiple pharmacological activities like anti-inflammatory, anti-tumor, and neuroprotective effects, Tan I has also been reported to improve insulin resistance in type 2 diabetes (Jung et al. 2020; Liu and Liu 2020; Wei et al. 2017). Earlier research has shown that Tan I has obvious anti-inflammatory activity and can be used as a potential anti-inflammatory drug (Wang et al. 2015; Yang et al. 2021). However, its anti-inflammatory mechanism and direct target remain to be further clarified.

In this study, we found that Tan I markedly suppressed the activation of NLRP3 inflammasome, but had no impact on NLRC4 or AIM2 inflammasome. Tan I inhibited NLRP3 inflammasome assembly by disrupting the interaction between NLRP3 and ASC and showed a significant protective effect against LPS-induced septic shock and NASH in mice. Collectively, our findings identify Tan I as an attractive inhibitor of NLRP3 inflammasome, providing a potential therapeutic candidate for NLRP3-mediated inflammatory diseases.

## Materials and methods

### Antibodies and chemicals

Tanshinone I (Tan I, HY-N0134), MCC950 (HY-12815 A), and nigericin (HY-127019) were from MedChemExpress. ATP, SiO_2_, Pam3CSK4, poly (I:C), poly (dA:dT), and ultrapure lipopolysaccharide (LPS) were from Invivogen. Phorbol 12-myristate 13-acetate (PMA, P8139) and DMSO (D2650) were from Sigma Aldrich. The Starfect high-efficiency transfection reagent (C101-10) was from GenStar. Antibodies were used as follows: anti-mouse IL-1β (1:1000, AF-401-NA, R&D), anti-mouse caspase-1 (1:1000, AG-20B-0042, Adipogen), anti-human cleaved IL-1β (1:2000, 12242, Cell Signaling Technology), anti-human caspase-1 (1:2000, 4199 S, Cell Signaling Technology), anti-NLRP3 (1:2000, AG-20B-0014, Adipogen), anti-ASC (1:1000, sc-22514-R, Santa Cruz), anti-Flag (1:2000, 20543-1-AP, Proteintech), and anti-GAPDH (1:5000, 60004-1-Ig, Proteintech).

### Cell culture

Primary mouse bone marrow-derived macrophages (BMDMs) were isolated from the femurs and tibias of C57BL/6, and cultured in DMEM complemented with 10% FBS, 1% penicillin/streptomycin (P/S) and 50 ng/ml mouse macrophage colony stimulating factor (M-CSF, HY-P7085, MedChemExpress), then cultured for 6–7 days. THP-1 cells were cultured in RPMI 1640 medium (10% FBS, 1% P/S). HEK-293T cells were cultured in DMEM (10% FBS, 1% P/S). All cell lines were grown in a 5% CO_2_ incubator at 37 °C.

### Cell viability assay

BMDMs were plated overnight, and then exposed to different concentrations of Tan I for 24 h. CellTiter-Glo® 2.0 Cell Viability Assay (G9241, Promega) was used to analyze the cell viability following the manufacturer’s instructions.

### Inflammasome activation assay

BMDMs (1.2 × 10^6^/ml) were plated overnight, culture medium was replaced with fresh media containing 50 ng/ml LPS or 400 ng/ml Pam3CSK4 for 4 h, then culture medium was replaced with Opti-MEM containing Tan I for 1 h before being stimulated with inflammasome agonists: nigericin (10 µM, 45 min), ATP (5 mM, 1 h), SiO_2_ (250 µg/ml, 6 h), Salmonella (200 ng/ml, 6 h); poly (dA:dT) (2 µg/ml), poly (I:C) (2 µg/ml), or ultra-LPS (1 µg/ml) was transfected into the cells for 6 h.

### Immunoblotting

Immunoblotting analyses were conducted as previously described (Huang et al. 2018). Supernatant was added with trichloroacetic acid to precipitate the protein. For cell lysis, cells were lysed in SDS loading buffer. The protein was separated by SDS-PAGE and transferred to PVDF, indicating primary antibodies were used for blots, then incubated with HRP-conjugated species-specific antibodies. Enhanced chemiluminescence was used to visualize the proteins.

### ASC oligomerization

The ASC oligomerization was carried out as previously described (Liu et al. 2021). Briefly, samples were lysed with Triton buffer (50 mM Tris-HCl [pH 7.5], 150 mM NaCl, 0.5% [*v/v*] Triton X-100) containing protease inhibitor cocktail (C0001, TargetMol) and centrifuged at 4 °C, 6000 g for 10 min. Then, the pellets were washed twice and resuspended in 200 µl ice-cold PBS, and disuccinimidyl suberate (DSS, 2 mM) was added for cross-linking at 37 °C for 30 min, followed by centrifugation at 6000 g for 10 min at 4 °C. The cross-linked pellets were resolved in SDS loading buffer and analyzed by immunoblotting.

### Immunoprecipitation assay

HEK-293T cells were plated overnight, followed by transfection with ASC, Flag-NLRP3 plasmids, and then incubated with 20 µM Tan I for 6 h. Subsequently, samples were lysed with NP-40 buffer (0.1% [*v/v*] Nonidet-P40, 50 mM Tris [pH 7.8], 10% [*v/v*] glycerol, 50 mM NaCl, and 5 mM EDTA) containing protease inhibitor cocktail, and centrifuged at 4 °C, 12,000 g for 15 min, supernatants were then incubated with anti-Flag M2 beads (A2220, MilporeSigma) for 6 h at 4 °C.

### Intracellular K^+^ measurement

K^+^ was measured as in the previous description (Shi et al. 2020). Briefly, cells were lysed by ultrapure HNO_3_, and boiled at 100 °C for 30 min. Intracellular K^+^ measurements were performed by inductively coupled plasma mass spectrometry.

### ROS measurement

The assay for ROS measurement was performed as described previously (Wang et al. 2020b). Samples were harvested and stained with MitoSOX (Invitrogen) for 15 min at 37 °C, washed with HBSS for 3 times, then analyzed by flow cytometry.

### Mice

C57BL/6 mice (8–10 weeks, 18–22 g) were obtained from SPF Biotechnology Co., Ltd. (Beijing, China). Mice were maintained under pathogen-free conditions; treatments were randomly allocated. Animal experiments were conducted by protocols approved by the Animal Care and Use Committee of the Fifth Medical Center of the Chinese PLA General Hospital.

### LPS-induced septic shock

For the septic shock model, 8-week-old female C57BL/6 mice were pretreated with vehicle, MCC950 (20 mg/kg, i.p.) or Tan I (10 mg/kg, 20 mg/kg, i.p.) (n = 10) for 2 h before injection of 20 mg/kg LPS intraperitoneally, mice were monitored for lethality for 3 days. To detect inflammatory cytokine production, mice were treated with Tan I (20 mg/kg, i.p.) for 2 h, then administered with 20 mg/kg LPS intraperitoneally, 4 h later, mice were euthanized, IL-1β in the plasma or peritoneal lavage fluid was measured by ELISA (R&D).

### Methionine-choline-deficient diet model

For induction of NASH, male C57BL/6 mice (8 weeks, 18-22 g) were fed a methionine-choline-deficient (MCD, 518,810, Dyets) diet, whereas the control groups received methionine and choline supplemented (MCS, 518,811, Dyets) diet, under the manufacturer’s instructions. The MCD and MCS fed mice were divided into groups (n = 6) that received vehicle, Tan I (20 mg/kg/d), or MCC950 (20 mg/kg/d) for five days, and then 40 mg/kg via gavage every other day for six weeks (Tan I or MCC950 was given starting when the mice were fed the MCD or MCS diet). Serum and liver were collected after mice were euthanized. Serum levels of AST and ALT (Nanjing jiancheng) were detected, while the pathological changes of the liver were detected by HE staining, Sirius red staining, and Masson staining.

### Statistics

For statistical analysis, GraphPad Prism 6 was utilized. For comparison of multiple groups, one-way ANOVA with Dunnett’s post hoc test or Sidak’s post hoc test was used, and unpaired Student’s *t*-text was used to evaluate differences between the two groups. All of the experimental data were presented as mean ± SEM. The difference was considered statistically significant at * *P* < 0.05; NS: not significant.

## Results

### Tan I potently inhibits NLRP3 inflammasome activation

**Fig. 1:**
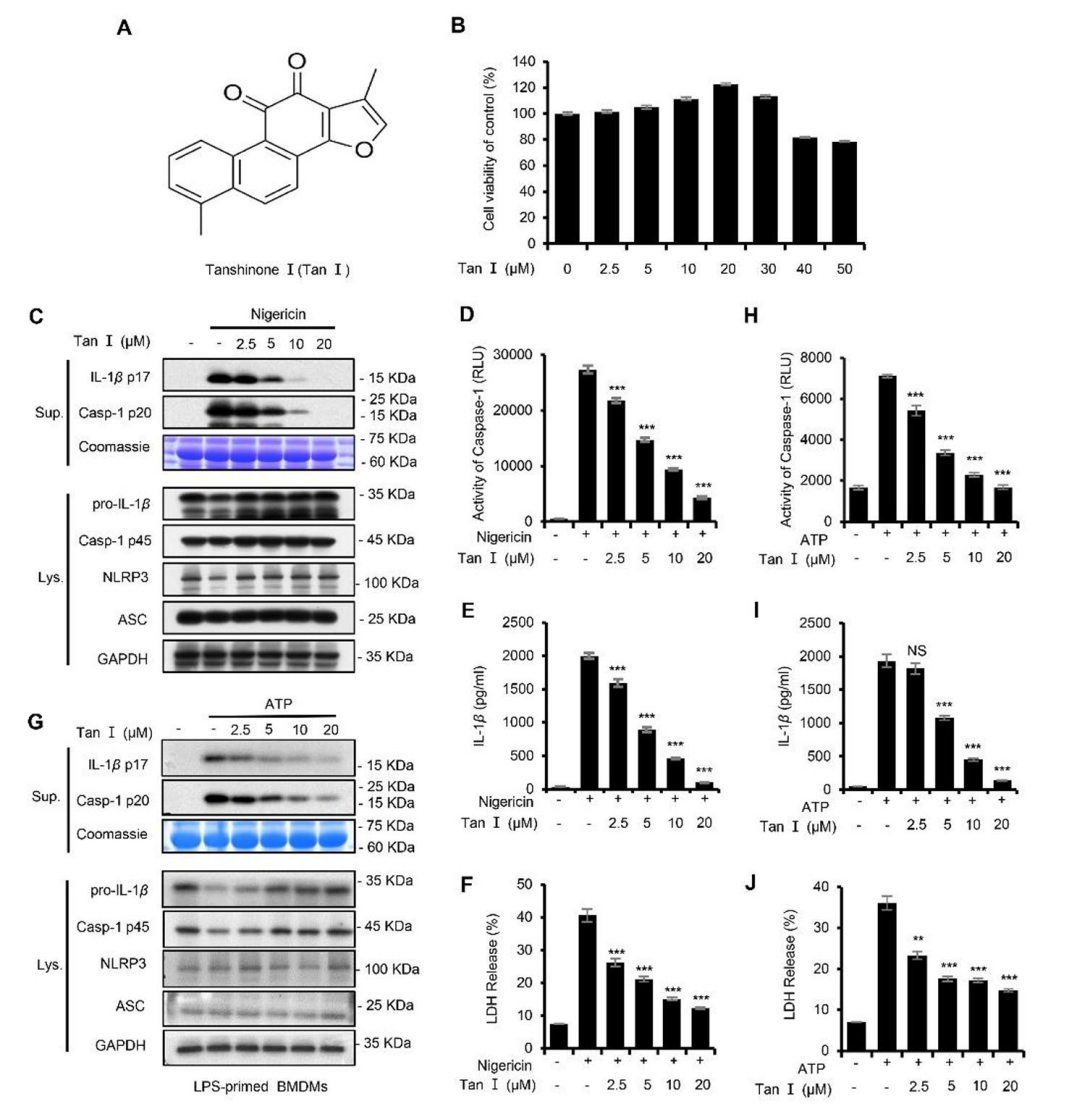
Tanshinone I (Tan I) suppressed NLRP3 inflammasome activation in BMDMs. (**A**) Tan I’s structure. (**B**) Cell viability of BMDMs treated with Tan I. (**C**-**J**) BMDMs were primed with LPS, then incubated with Tan I and stimulated with nigericin or ATP, cleaved caspase-1(p20) and mature IL-1β (p17) in supernatant (Sup.) were assessed by immunoblotting (**C**, **G**), caspase-1 activity (**D**, **H**), IL-1β secretion (**E**, **I**), and LDH release (**F**, **J**) in the supernatant were detected. Data are shown as mean ± SEM from triplicates, **P < 0.01, ***P < 0.001, NS: not significant (one-way ANOVA with Dunnett’s post hoc test)

Before exploring the impact of Tan I (Fig. 1A) on NLRP3 inflammasome activation, its effect on cell viability was measured. Bone-marrow derived macrophages (BMDMs) were exposed to different doses of Tan I for 24 h. Data showed that Tan I was not toxic at concentrations below 30 µM (Fig. 1B). NLRP3 inflammasome mediates caspase-1 cleavage and IL-1β maturation, and pyroptosis (Liu et al. 2016). Thus, we tested whether Tan I could inhibit the maturation and secretion of IL-1β, caspase-1 activity, and LDH release (a marker for cell death (Shi et al. 2020), triggered by NLRP3 inflammasome agonists. We illustrated that Tan I inhibited caspase-1 cleavage (Fig. 1C and D), IL-1β maturation (Fig. 1C and E), and LDH release (Fig. 1F) induced by nigericin in a dose-responsive manner. Besides, Tan I also prevented ATP-induced NLRP3 inflammasome activation (Fig. 1, G-J). Moreover, our results suggested that Tan I inhibited caspase-1 cleavage as well as IL-1β maturation triggered by NLRP3 agonist nigericin in THP-1 cells, the human monocyte-like cell line (Supplemental Fig. 1, A-C). In general, our results suggested that Tan I suppressed NLRP3 inflammasome activation.

### Tan I had no impact on NLRC4 or AIM2 inflammasome activation

**Fig. 2:**
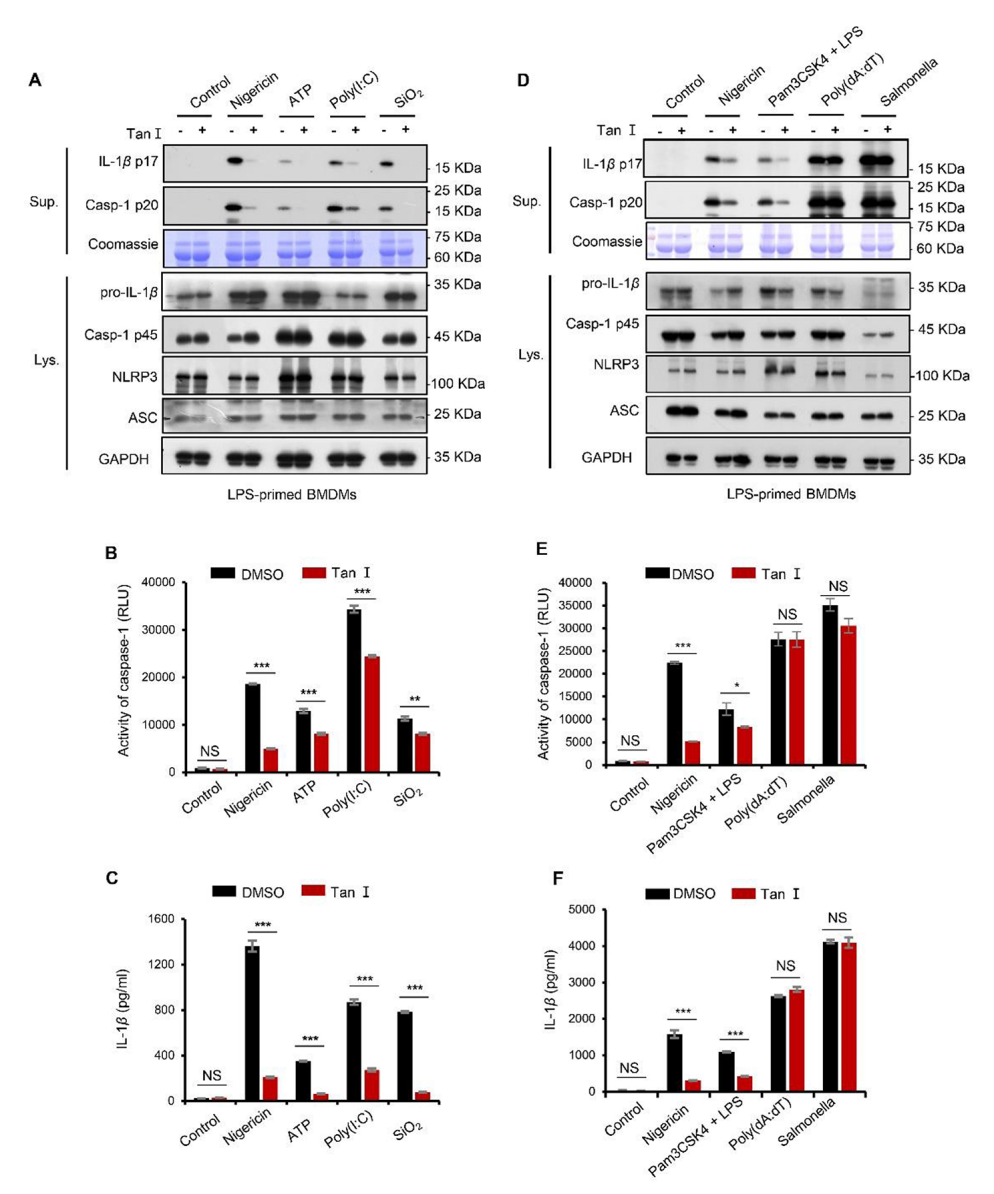
Tan I had no impact on NLRC4 or AIM2 inflammasome activation. (**A**–**C**) LPS-primed BMDMs were incubated with or without Tan I (20 µM), followed by the indicated stimuli. Cleaved caspase-1(p20) and mature IL-1β (p17) in supernatant were assessed by immunoblotting (**A**), activity of caspase-1 (**B**) and IL-1β secretion (**C**) in the supernatants were analyzed. (**D**-**F**) LPS-primed BMDMs were incubated with or without Tan I (20 µM), followed by treatment with nigericin, poly(dA:dT) or Salmonella, or Pam3CSK4-primed BMDMs were treated with Tan I followed by transfection of LPS, cleaved caspase-1(p20) and mature IL-1β (p17) were assessed by immunoblotting (**D**), activity of caspase-1 (**E**) and IL-1β secretion (**F**) in supernatants were evaluated. Data are shown as mean ± SEM from triplicates, *P < 0.05, **P < 0.01, ***P < 0.001, NS: not significant (unpaired Student’s t test)

We further studied Tan I’s impact on other stimuli-induced NLRP3 inflammasome activation. LPS-primed BMDMs were treated with Tan I before being stimulated with poly (I: C), SiO_2_, or ATP. Similarly, we found that Tan I obviously reduced caspase-1 cleavage and IL-1β maturation (Fig. 2, A-C). Additionally, our results showed that Tan I blocked caspase-1 cleavage and IL-1β maturation induced by LPS transfection in Pam3CSK4-primed BMDMs (Fig. 2, D-F), demonstrating the suppression effect of Tan I on non-canonical NLRP3 inflammasome activation. Results showed that Tan I is a potent inhibitor of the NLRP3 inflammasome.

AIM2 and NLRC4 inflammasomes also mediate caspase-1 activation (Song et al. 2018). Therefore, we examined whether Tan I could prevent AIM2 or NLRC4 inflammasome activation. After priming, BMDMs were infected with *Salmonella typhimurium* or transfected with poly (dA: dT) to activate NLRC4 or AIM2 inflammasome, respectively (Bauer and Rauch 2020; Hornung et al. 2009). Of note, our results proved that Tan I blocked neither caspase-1 activation nor IL-1β maturation triggered by *Salmonella typhimurium* or poly (dA: dT) (Fig. 2, D-F). These data confirmed that Tan I specifically inhibits NLRP3 inflammasome activation, but not AIM2 or NLRC4 inflammasome.

### Tan I suppressed ASC oligomerization

**Fig. 3:**
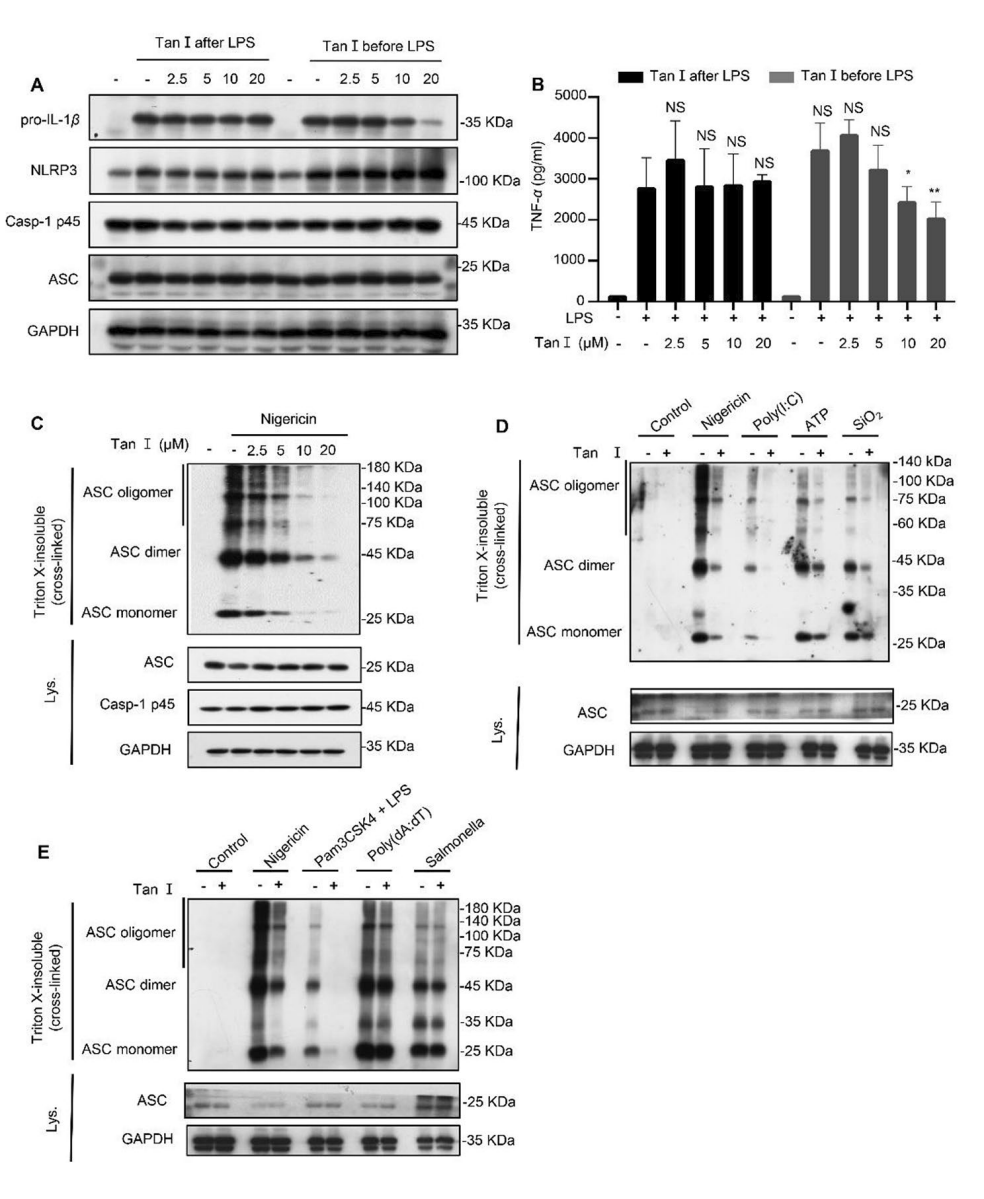
Tan I suppressed ASC oligomerization. (**A** and **B**) BMDMs were cultured with LPS for 4 h, then incubated with DMSO or Tan I for 1 h, or BMDMs were first incubated with Tan I for 1 h, followed by LPS treatment for 4 h, protein level of NLRP3 and pro-IL-1β was determined by immunoblotting (**A**), ELISA was used to detect TNF-α secretion (**B**). (**C**-**E**) Immunoblotting of cross-linked pellets from LPS-primed BMDMs treated with Tan I before being stimulated by the indicated agonists. Data are shown as mean ± SEM from triplicates, *P < 0.05, **P < 0.01, NS: not significant (one-way ANOVA with Dunnett’s post hoc test)

Previous research revealed that Tan I inhibits NF-κB activation (Wang et al. 2015; Yang et al. 2021), which mediates the expression of pro-1 L-1β and NLRP3 during LPS priming (Jo et al. 2016). Therefore, we tested whether Tan I influenced the generation of NLRP3 and pro-IL-1β mediated by LPS. BMDMs were exposed to Tan I before or after LPS stimulation. We found that Tan I inhibited the production of pro-IL-1β and TNF-α when pre-treated with Tan I for one hour before LPS stimulation (Fig. 3, A and B), but in the condition that LPS-primed BMDMs were treated with Tan I, Tan I did not affect pro-IL-1β expression. However, it still affected IL-1β secretion induced by NLRP3 agonists (Fig. 2A), indicating that Tan I indeed affects the activation step of NLRP3 inflammasome.

Then, the mechanism underlying Tan I’s effect on NLRP3 inflammasome was studied. ASC oligomerization acts an indispensable role in caspase-1 cleavage during inflammasome activation (Song and Li 2018). We found that Tan I inhibited the oligomerization of ASC induced by nigericin (Fig. 3C) dose-dependently. Further research showed that ASC oligomerization triggered by other NLRP3 agonists was also inhibited by Tan I (Fig. 3, D and E). In addition, Tan I had no influence on ASC oligomerization during NLRC4 or AIM2 inflammasome activation (Fig. 3E).

### Tan I restrained NLRP3 inflammasome activation by disrupting NLRP3-ASC interaction

**Fig. 4:**
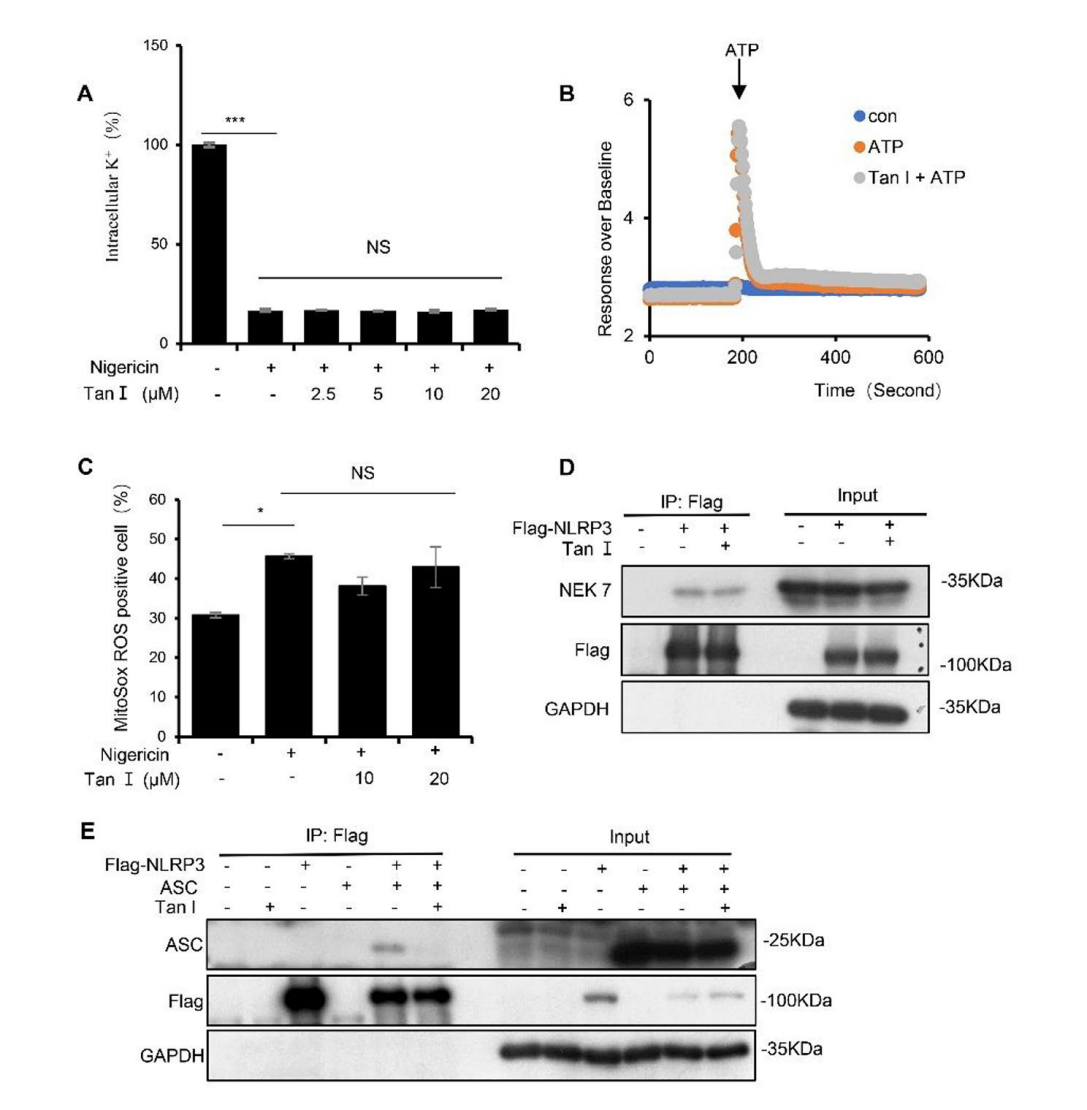
Tan I inhibited NLRP3 inflammasome by disrupting the association between NLRP3 and ASC. (**A**) BMDMs were primed with LPS followed by Tan I incubation, and then treated with nigericin, the efflux of potassium was measured. (**B**) The mobilization of Ca^2+^ in LPS-primed BMDMs treated with Tan I (or not) followed by ATP for the indicated time was assessed. (**C**) LPS-primed BMDMs were cultured in the presence or absence of Tan I and then stimulated with nigericin, then stained with MitoSox, mtROS was analyzed by flow cytometry. (**D**, **E**) Immunoprecipitation analysis of the interaction among NLRP3, NEK7, ASC in HEK-293T, in the presence of Tan I (20 µM) or not. Data are shown as mean ± SEM from triplicates, *P < 0.05, ***P < 0.001, NS: not significant (one-way ANOVA with Sidak’s post hoc test)

Next, we tested whether Tan I affected the upstream signaling events of NLRP3 inflammasome activation, including K^+^ efflux, Ca^2+^ flux, as well as mitochondrial reactive oxygen species (ROS) production (Muñoz-Planillo et al. 2013; Piccini et al. 2008; Zhou et al. 2011). Intracellular potassium was significantly reduced after nigericin treatment, but this effect was not affected by Tan I (Fig. 4A). NLRP3 agonists, such as nigericin and ATP, have been reported to trigger Ca^2+^ mobilization, blocking Ca^2+^ signaling would inhibit NLRP3 inflammasome activation (Lee et al. 2012; Murakami et al. 2012). Herein, LPS-primed BMDMs were stimulated with ATP and Ca^2+^ mobilization was observed, but this mobilization was not affected by Tan I (Fig. 4B). Moreover, mtROS generated by mitochondrial damage is considered to be another upstream signaling event in NLRP3 inflammasome activation (Zhou et al. 2011). Our results demonstrated that Tan I did not influence the mitochondrial ROS production stimulated by nigericin (Fig. 4C).

Subsequently, investigations were conducted into Tan I’s impact on NLRP3 inflammasome assembly. NEK7 is a vital regulator of NLRP3 inflammasome and the interaction between NLRP3 and NEK7 is essential for NLRP3 inflammasome activation (He et al. 2016). Once activated, NLRP3 recruits ASC and pro-caspase-1 to form the inflammasome (Shi et al. 2016). So, we further explored whether Tan I could prevent the interaction between the core proteins of NLRP3 inflammasome. Our data suggested that Tan I treatment suppressed the interaction between ASC and NLRP3, but not NLRP3 and NEK7 (Fig. 4, D and E). Thus, our data uncovered that Tan I suppressed NLRP3 inflammasome activation via blocking NLRP3-ASC interaction.

### Tan I relieved LPS-induced septic shock

**Fig. 5:**
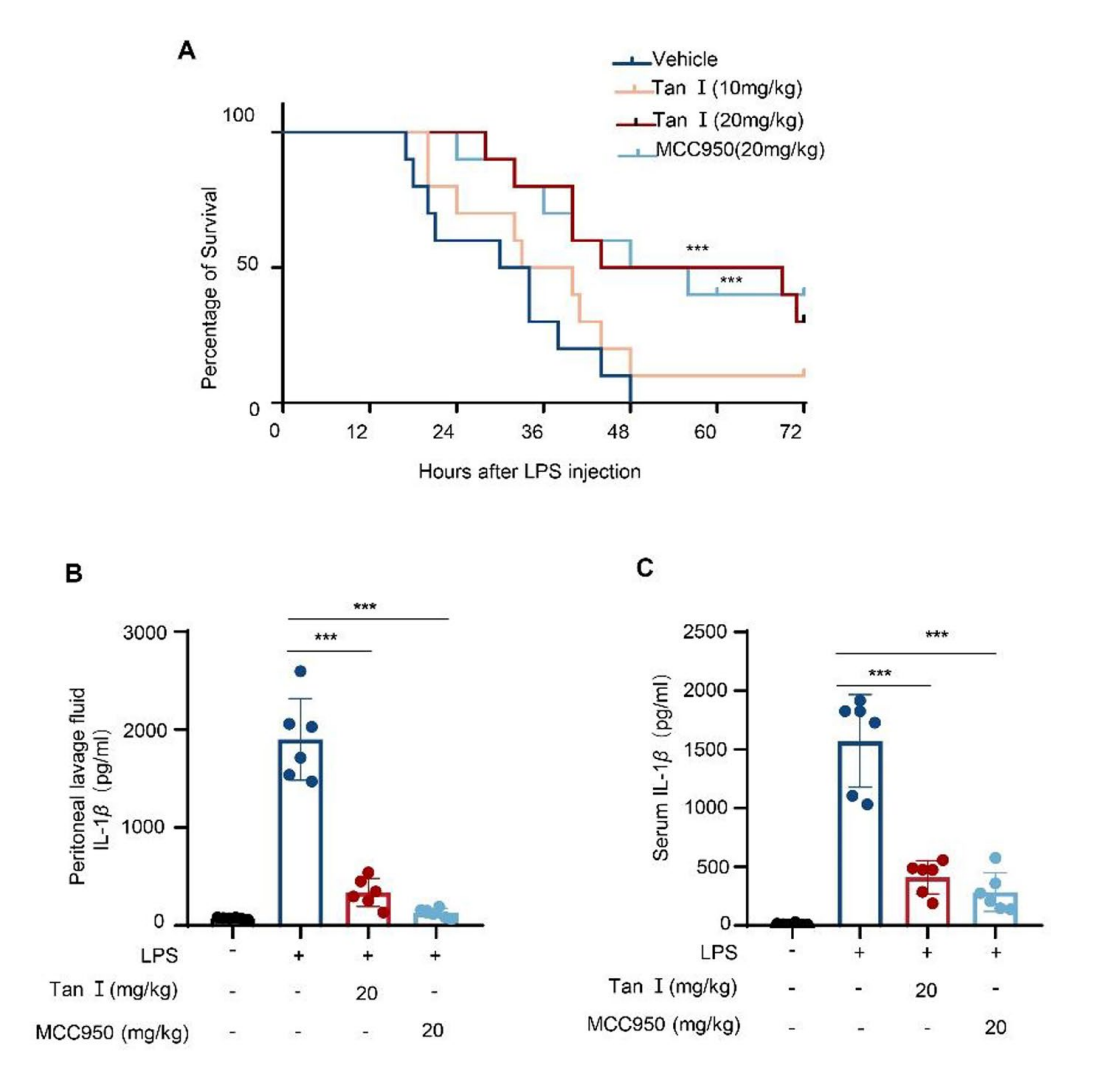
Tan I relieved LPS-induced septic shock and inhibited NLRP3 inflammasome activation in vivo. (**A**) Female mice (8 weeks) were intraperitoneally (i.p.) injected with vehicle, MCC950 (20 mg/kg), or Tan I (10, 20 mg/kg) respectively, 2 h later, administered with LPS (20 mg/kg, i.p.), then monitored for 72 h (n = 10). (**B**, **C**) Mice were injected intraperitoneally with 20 mg/kg Tan I, 20 mg/kg MCC950 for 2 h, followed by an injection of 20 mg/kg LPS for 4 h (n = 6). The levels of IL-1β in peritoneal lavage fluid (**B**) and serum (**C**) were analyzed by ELISA. Data are shown as mean ± SEM, ***P < 0.001 (log-rank test (A), one-way ANOVA with Dunnett’s post hoc test (**B** and **C**))

Moreover, the influence of Tan I on NLRP3 inflammasome activation was evaluated in vivo. Previous studies have reported that LPS injected intraperitoneally induces NLRP3-dependent IL-1β secretion and septic shock in mice (Mao et al. 2013). In our study, mice were injected with LPS intraperitoneally after pre-treatment with Tan I or MCC950, a reported inhibitor of NLRP3 (Wu et al. 2020), the survival was observed. In our study, Tan I dose-dependently protected mice from septic shock (Fig. 5A). To test Tan I’s influence on IL-1β secretion in vivo, pretreated mice with Tan I or MCC950, followed by injection with LPS. Four hours later, the samples were collected and the level of IL-1β in peritoneal lavage fluid and serum was determined. The data showed that LPS injection induced the production of IL-1β, which was suppressed by Tan I, and it had a similar effect compared to MCC950 (Fig. 5, B and C). Collectively, Tan I protected mice from septic shock and inhibited NLRP3 inflammasome activation in vivo.

### Tan I ameliorated non-alcoholic steatohepatitis in mice

**Fig. 6:**
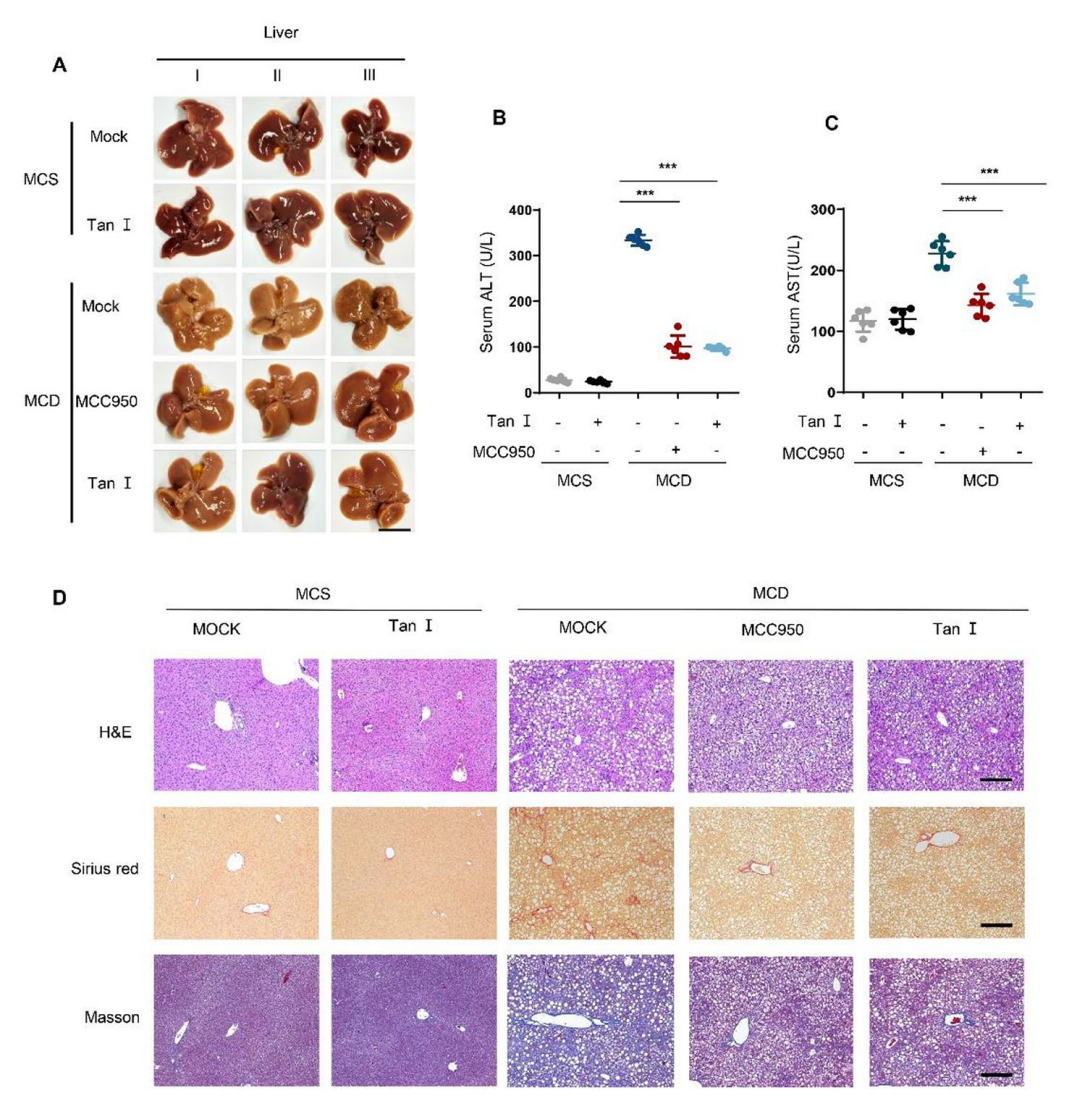
Tan I ameliorated non-alcoholic steatohepatitis in mice. (**A**-**D**) Male C57BL/6 mice (8 weeks, 18-22 g) were fed MCD diet, while the control groups received MCS diet. The MCD and MCS fed mice were divided into groups (n = 6) that received vehicle, Tan I (20 mg/kg), or MCC950 (20 mg/kg) every day for 5 days, and subsequently 40 mg/kg via gavage every other day for six weeks. The liver morphology of mice in different treatment groups was shown, scale bar: 1 cm (**A**). Serum ALT and AST levels were determined by ELISA (**B** and **C**). Liver sections were stained with HE, Sirius Red, and Masson, scale bar: 200 μm (**D**). Data are shown as mean ± SEM, ***P < 0.001 (one-way ANOVA with Dunnett’s post hoc test)

NLRP3 inflammasome is reported to play a crucial role in NASH (Mridha et al. 2017). Since 2000, the methionine and choline deficient diet (MCD) model has been widely utilized to produce severe steatohepatitis and fibrosis, that mimics the pathogenesis of human NASH (Ioannou et al. 2013; Leclercq et al. 2000). The histological abnormalities of NASH, a frequent liver ailment, resemble those of alcoholic steatohepatitis. Steatosis, hepatocyte injury, a mixed inflammatory lobular infiltration, and fibrosis are among the histological characteristics. Mice acquired severe steatohepatitis quickly and consistently under the MCD dietary regimen. Steatosis, inflammatory infiltration, hepatic necrosis, and fibrosis are the pathologies that are typical of NASH in humans (Ludwig et al. 1980; Powell et al. 1990). In recent years, a growing number of studies have confirmed the relationship between NLRP3 and NASH, and blockade of NLRP3 inflammasome activation can reduce liver inflammation and fibrosis as well as improve NASH pathology (Calcagno et al. 2022; Mridha et al. 2017). Therefore, we tested the protective effect of Tan I in MCD-induced NASH in mice, and the data suggested that administration of Tan I or MCC950 reversed the liver morphology in the NASH mice, accompanied the reduction of serum AST and ALT induced by MCD diet (Fig. 6, A-C). Furthermore, hepatic histopathological analysis was performed with HE, Sirius red, and Masson staining. Liver fibrosis, fatty vacuoles, and inflammatory cell infiltration were observed in the NASH mice (MCD), while Tan I treatment alleviated these histopathological changes (Fig. 6D, Supplemental Fig. 2, A and B). Moreover, we detected the expression of F4/80 in the liver tissues and found that the expression of F4/80^+^ was reduced in Tan I and MCC950 treated NASH mice (Supplemental Fig. 2, C and D). To determine whether Tan I blocked the activation of NLRP3 inflammasome in NASH mice, immunoblotting was used to detect the expression of active caspase-1 in mouse liver tissues. Our results showed that caspase-1 activation in the liver of mice fed MCD diets was inhibited by Tan I or MCC950 (Supplemental Fig. 2E). Taken together, these data confirmed that Tan I exhibited a protective effect in the mouse model of NASH.

## Discussion

NLRP3 inflammasome mediates the development of many inflammatory diseases, like NASH, acute lung injury, gout, type 2 diabetes, and preeclampsia (Guan et al. 2022; Mangan et al. 2018; Szabo and Petrasek 2015; Zhao et al. 2021; Zhu and Liu 2022). Therefore, targeting NLRP3 inflammasome is considered a prospective strategy. Herein, we report that the main active ingredient of *Salvia miltiorrhiza*, Tan I specifically inhibits NLRP3 inflammasome activation both in vitro and in vivo, and exhibits an obviously protective effect in mouse models of NLRP3-mediated inflammatory diseases, indicating that Tan I might be a potent therapeutic drug candidate.

In our study, we found that Tan I did not affect the upstream signaling events of NLRP3 inflammasome activation, including K^+^efflux, Ca^2+^flux, as well as ROS production. Meanwhile, our results illustrated that Tan I had no impact on NLRP3-NEK7 interaction but inhibited NLRP3-ASC interaction, suggesting Tan I blocked NLRP3 inflammasome activation via disrupting ASC recruitment to NLRP3, but the direct target needs further study. In addition, whether Tan I inhibits NLRP3 interaction with ASC in vivo needs further investigation.

Tan I has been reported to inhibit NF-κB activation (Wang et al. 2015), in our study, we found that Tan I inhibited NF-κB-mediated pro-IL-1β expression in BMDMs when pre-treated with Tan I followed by LPS priming. We noticed that in this contest Tan I had no effect on NLRP3 expression, this may be because pro-IL-1β is more sensitive to the activation of NF-κB, or due to the differential regulatory effect of Tan I on the production of NLRP3 and pro-IL-1β.

Furthermore, we also found that Tan I had a significant beneficial effect on NLRP3 inflammasome-related diseases like septic shock and NASH in mice. Meanwhile, Tan I also suppressed IL-1β secretion in LPS-induced septic shock, suggesting Tan I’s inhibitory effect on NLRP3 inflammasome activation in vivo. NASH has become one of the important causes of liver cirrhosis and liver cancer, but currently there is no effective treatment. In our study, we found that Tan I could alleviate pathologic changes such as hepatic steatosis, inflammatory cell infiltration and liver fibrosis in MCD-induced NASH mice. This suggests that Tan I may be a promising therapeutic candidate for NASH. In addition, previous research reported that Tan I reduced insulin resistance in type 2 diabetes rats (Wei et al. 2017) as well as osteoarthritis in mice (Wang et al. 2019a), NLRP3 inflammasome activation also act as a key role in the progression of type 2 diabetes as well as osteoarthritis. Whether Tan I ameliorates the progression of these diseases via its inhibitory effect on NLRP3 inflammasome requires future studies.

Previous studies revealed that tanshinones, the main component of *Salvia miltiorrhiza*, have been reported to have anti-inflammatory effects and may be the key component for its anti-inflammatory effects (Jiang et al. 2019; Liu et al. 2022; Wang et al. 2020a). In addition, Tanshinone IIA and Dihydrotanshinone I have been reported to inhibit the activation of NLRP3 inflammasome and protect against NLRP3-related diseases (Gao et al. 2019; Li et al. 2022c; Wei et al. 2021; Xu et al. 2022). A recent study found that 15 tanshinones (isolated from *Salvia miltiorrhiza*) including Tan I inhibited IL-1β secretion, but did not further demonstrate the specificity of the effect of Tan I on NLRP3 inflammasome activation or the mechanism of action. Whether Tan I is protective against NLRP3-mediated diseases is not yet clarified (Yue et al. 2021). Our study clarified the regulatory effect of Tan I on NLRP3 inflammasome activation both in vitro and in vivo as well as the detailed mechanism, benefiting the understanding of the pharmacological effect of Tan I.

## Conclusions

In summary, we demonstrated that Tan I specifically inhibited NLRP3 inflammasome activation via targeting the ASC and NLRP3 complex. In vivo studies have shown that Tan I relieves LPS-induced septic shock and NASH in mice. Altogether, Tan I may be developed as a potent therapeutic drug candidate for the treatment of NLRP3 inflammasome-mediated diseases.

## Electronic supplementary material

Below is the link to the electronic supplementary material.

Supplementary Material 1

Supplementary Material 2

Supplementary Material 3

Supplementary Material 4

## Data Availability

The datasets used and/or analyzed during the current study are available from the corresponding author on reasonable request.

## Abbreviations

Tan I: tanshinone I
BMDMs: bone marrow-derived macrophages
NLRP3: NOD-like receptor thermal protein domain associated protein 3
ASC: apoptosis-associated speck-like protein
NASH: non-alcoholic steatohepatitis
LPS: Lipopolysaccharide
