# Supplementary figures and images for: Tanshinone I specifically suppresses NLRP3 inflammasome activation by disrupting the association of NLRP3 and ASC
Source: Mol Med. 2023 Jul 3;29:84. doi: 10.1186/s10020-023-00671-0 (PMC10318668; doi:10.1186/s10020-023-00671-0)

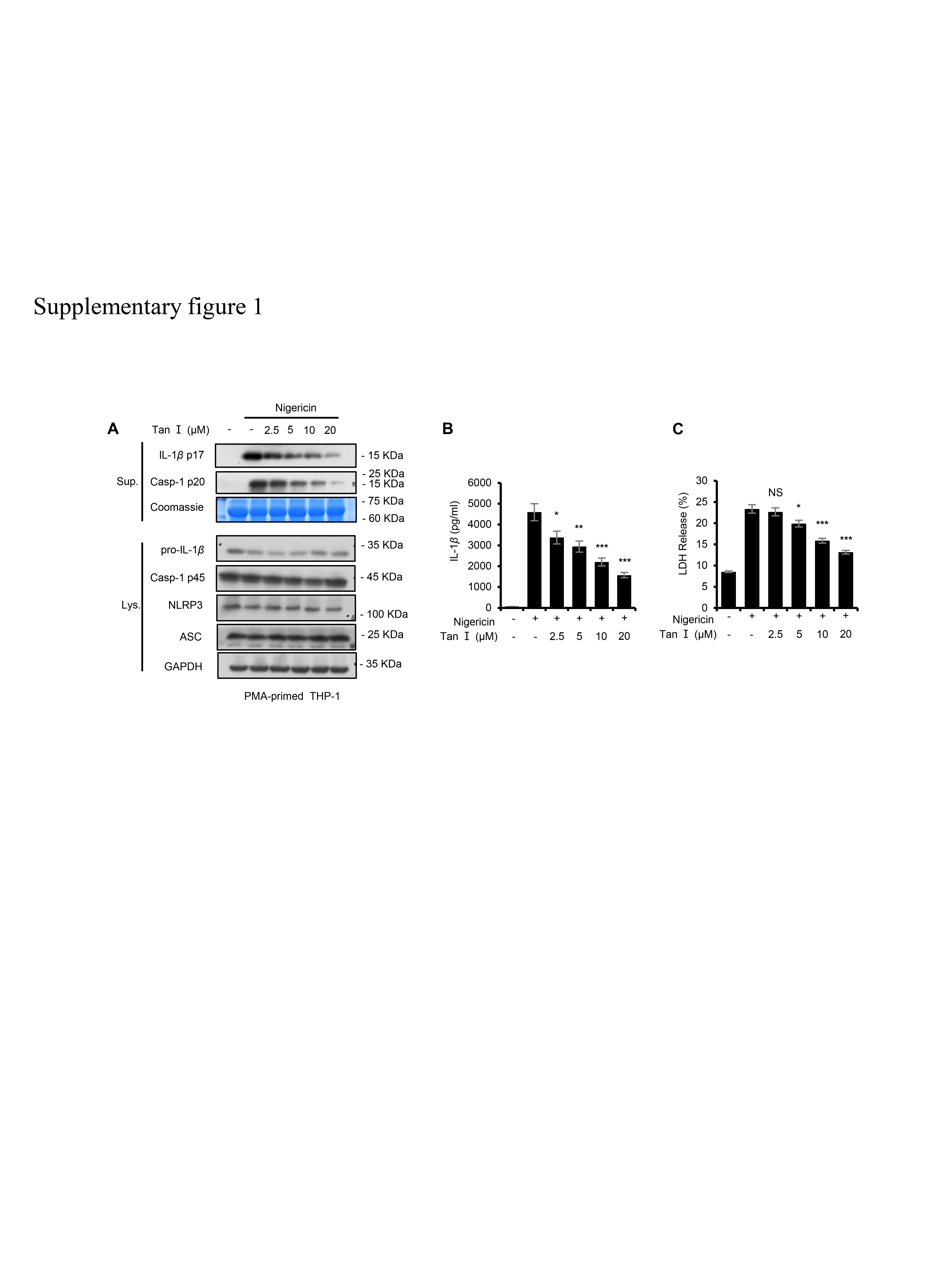

Supplement: Supplementary file 1 — Supplementary Material 1 [file 10020_2023_671_MOESM1_ESM.jpg]

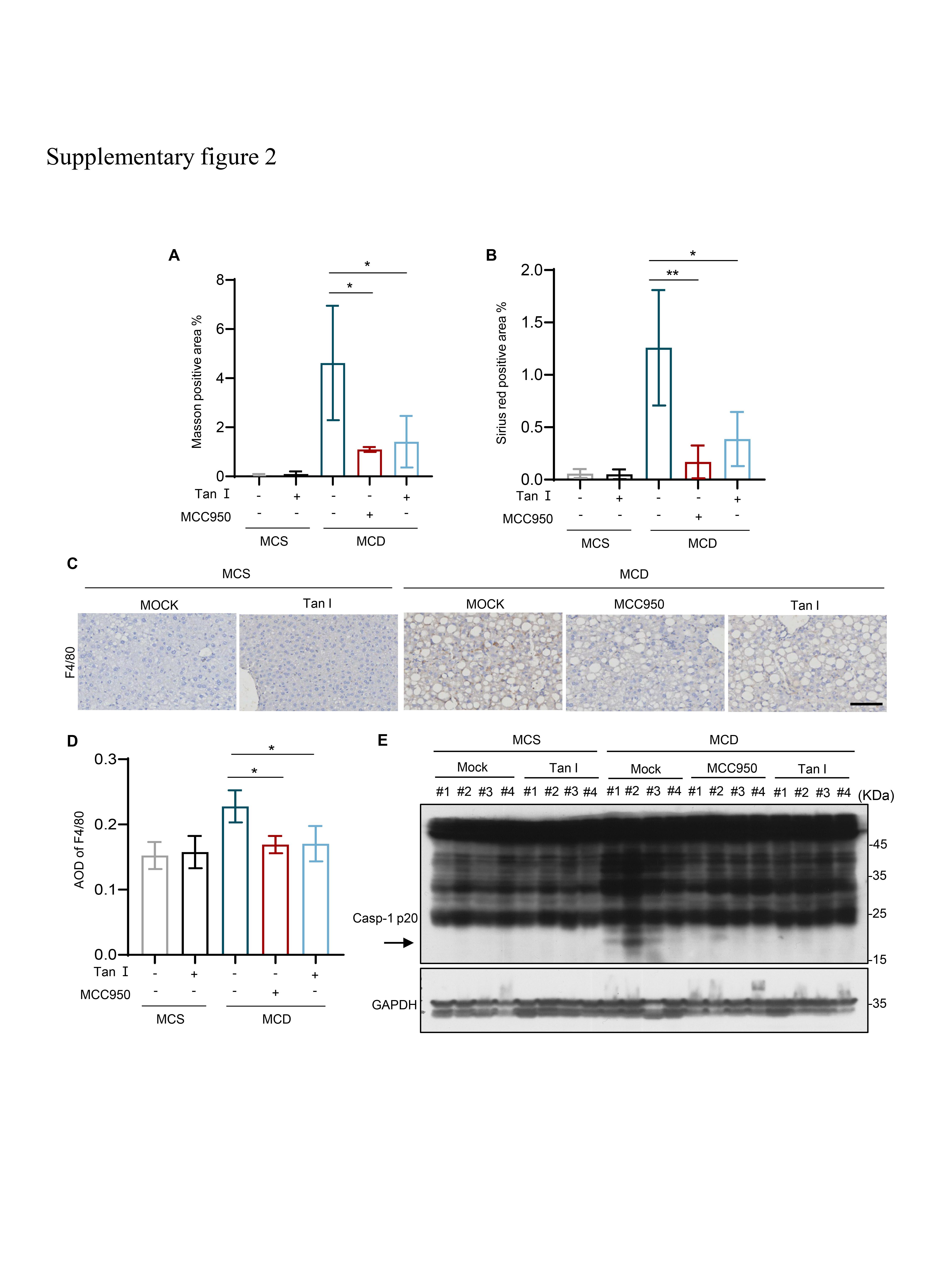

Supplement: Supplementary file 2 — Supplementary Material 2 [file 10020_2023_671_MOESM2_ESM.jpg]

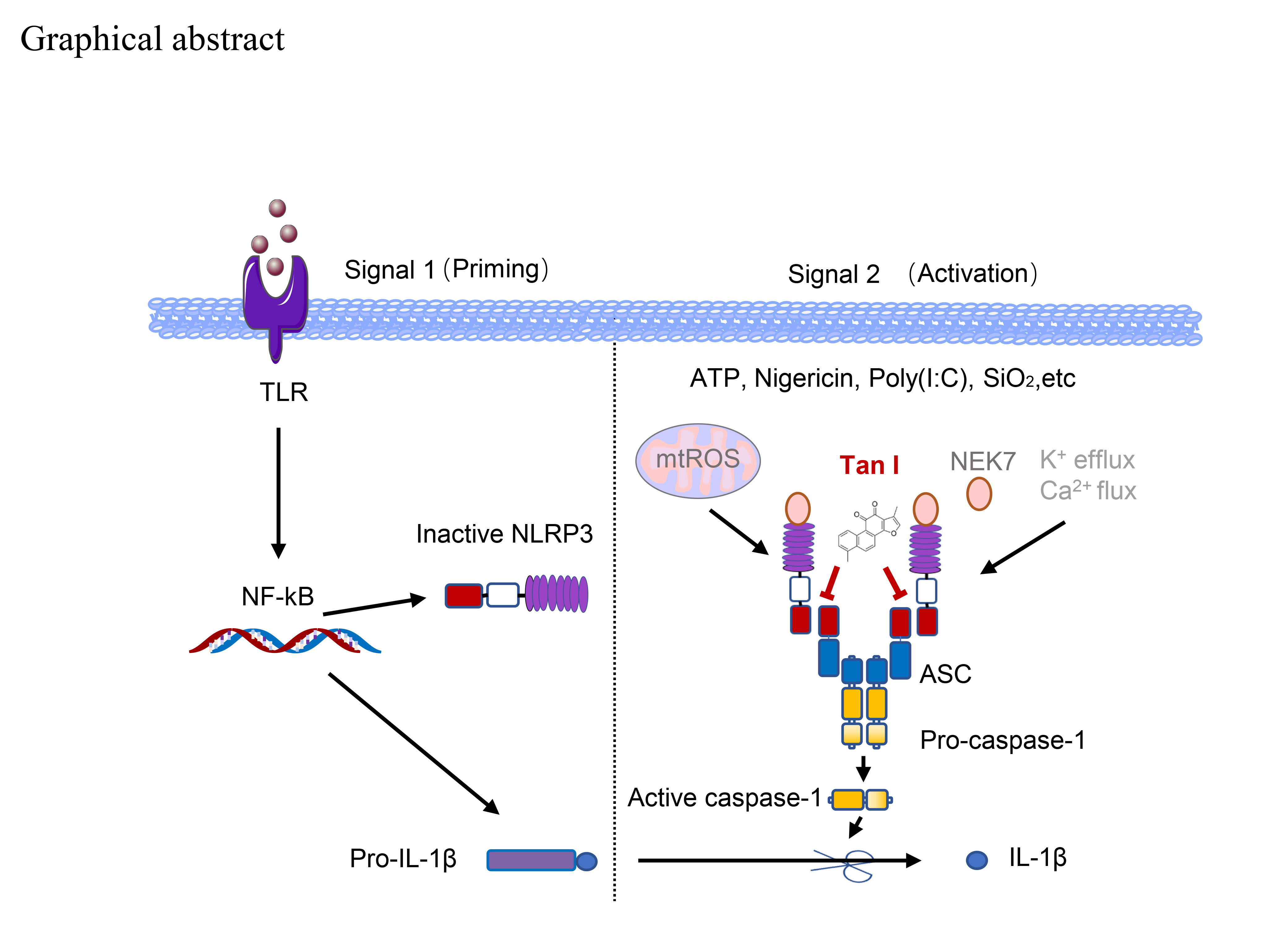

Supplement: Supplementary file 4 — Supplementary Material 4 [file 10020_2023_671_MOESM4_ESM.jpg]
