# Supplementary material for: Tanshinone I specifically suppresses NLRP3 inflammasome activation by disrupting the association of NLRP3 and ASC
Source: Mol Med. 2023 Jul 3;29:84. doi: 10.1186/s10020-023-00671-0 (PMC10318668; doi:10.1186/s10020-023-00671-0)
Supplement: Supplementary file 3 — Supplementary Material 3 [file 10020_2023_671_MOESM3_ESM.docx]

**Supplementary material**


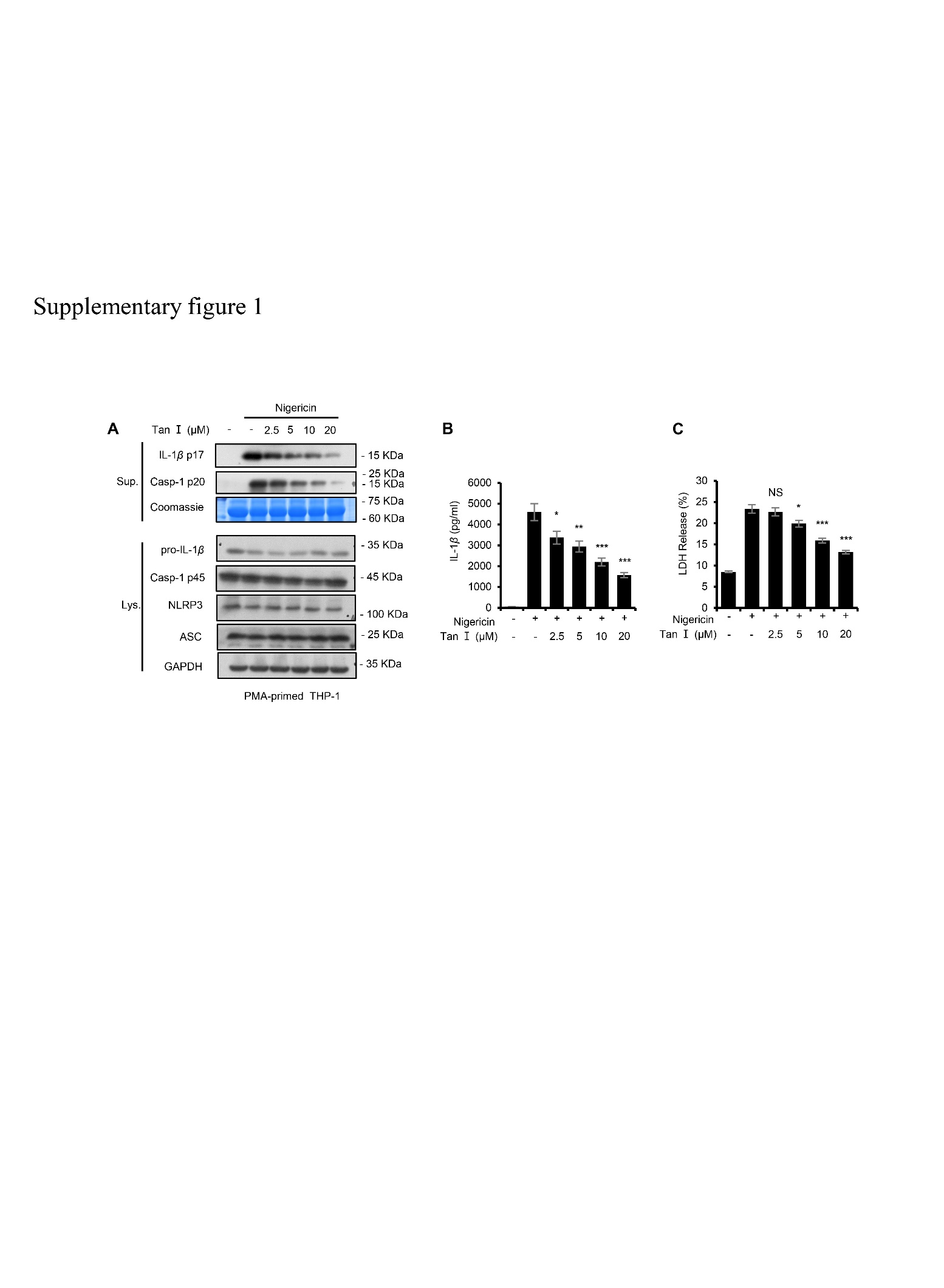


**Supplementary figure 1. Tan I inhibited NLRP3 inflammasome activation in THP-1.** (A-C) PMA-primed THP-1 cells were exposed to Tan I before stimulation with nigericin, caspase-1 p20 and IL-1β p17 in supernatant were assessed by immunoblotting (A), IL-1β secretion (B) and LDH release (C) in supernatant were detected. Data are shown as mean ± SEM from triplicates, **P* <0.05, ***P* <0.01, ****P* <0.001, NS: not significant (one-way ANOVA with Dunnett’s post hoc test).


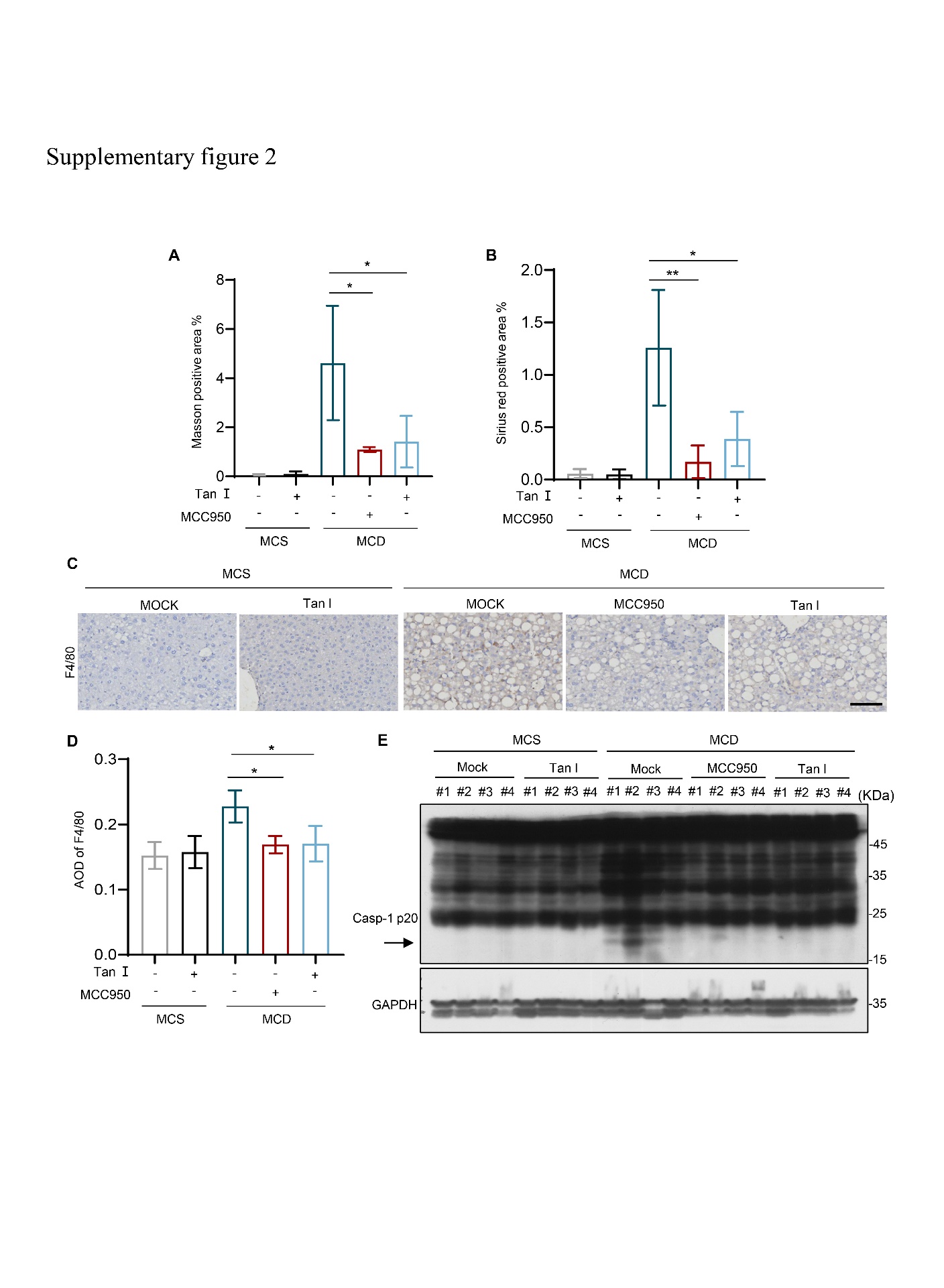


**Supplementary figure 2. Tan I ameliorated non-alcoholic steatohepatitis in mice.** (A) Statistical analysis of Masson positive area; (B) Statistical analysis of Sirius red positive area. (C) Representative images for immunohistochemical staining of F4/80 in the liver of NASH mice, scale bar: 100 μm; (D) Quantitative analysis of F4/80 positive areas, n=3. (E) Representative immunoblotting analysis of active caspase-1 in liver tissues, n=4; **P* <0.05, ***P* <0.01, ****P* <0.001, NS: not significant (one-way ANOVA with Dunnett’s post hoc test).
